# A Novel Lytic Podovirus AP-20-A Infecting *Sinorhizobium meliloti*: Mosaic Genome with Cross-Phylum Homology and Implications for Inoculant Establishment

**DOI:** 10.3390/ijms27125515

**Published:** 2026-06-18

**Authors:** Alexandra P. Kozlova, Marina L. Roumiantseva, Alla S. Saksaganskaia, Maria E. Vladimirova, Victoria S. Muntyan, Maria K. Gorbunova, Andrey N. Gorshkov

**Affiliations:** 1 Laboratory of Genetics and Selection of Microorganisms, Federal State Budget Scientific Institution All-Russia Research Institute for Agricultural Microbiology (FSBSI ARRIAM), 196608 Saint Petersburg, Russia; a.kozlova@arriam.ru (A.P.K.); allasaksaganskaya@arriam.ru (A.S.S.); mariiacherkasova@arriam.ru (M.E.V.); vucovar@arriam.ru (V.S.M.); m.gorbunova@arriam.ru (M.K.G.); 2 Institute of Biomedical Systems and Biotechnology, Graduate School of Biotechnology and Food Science, Peter the Great St. Petersburg Polytechnic University (SPbPU), Polytechnicheskaya, 29, 195251 Saint Petersburg, Russia; 3 Smorodintsev Research Institute of Influenza, Ministry of Health of the Russian Federation, 197376 Saint Petersburg, Russia; angorsh@yahoo.com

**Keywords:** *Sinorhizobium* phage, podovirus-morphotype, cross-phylum horizontal gene transfer (HGT), *Bacillota* (formerly *Firmicutes*) *Paenibacillus*, MoTad2 anti-defense system, AbiE system, ecological filter, inoculant vulnerability

## Abstract

This study characterizes AP-20-A, a lytic podovirus infecting *Sinorhizobium meliloti*, isolated from agricultural chernozem. Its 49.4 kbp genome shows negligible intergenomic similarity with known rhizobiophages (<2%). Core structural proteins—the major capsid protein (MCP) and terminase large subunit (TerL)—show closest homology to podoviruses infecting *Paenibacillus*, rather than to alphaproteobacterial viruses, suggesting cross-phylum horizontal gene transfer. This exchange is ecologically plausible, as *Paenibacillus* and *Sinorhizobium* co-exist in the rhizosphere. Over 63% of predicted proteins are functionally uncharacterized, with structural homologs detected in bacteria, archaea, and eukaryotes. We report the first identification in a rhizobiophage of a Tad2-like domain, predicted to block the bacterial Thoeris type II anti-phage defense. AP-20-A infected 56% of native *S. meliloti* strains; agrocenose isolates showed higher resistance than phytocenose isolates, evidence of local co-evolution. Among susceptible strains, 60% entered putative pseudolysogeny (with one strain exhibiting growth stimulation), whereas a symbiotically elite inoculant strain was completely lysed within hours. Some host strains carry additional AbiE systems; whether these independent defense–counterdefense layers interact during infection remains unknown. We conclude that resident phages represent a selective force that can disrupt inoculant establishment, underscoring the need to integrate soil virome assessment into agricultural microbiome management.

## 1. Introduction

Bacteriophages, as natural foes of bacteria and active participants in biogeochemical cycles, represent a powerful tool for managing microbial communities. Their lytic activity not only regulates bacterial populations but also contributes to the carbon cycle by enriching the soil with organic matter [1,2]. In agroecosystems, this property is central to biocontrol: the application of phages allows for the targeted suppression of phytopathogens and shifts the balance of soil microbiology towards beneficial bacteria, thereby modulating the composition of the soil microbiota without harming the environment [3]. High specificity, lack of phytotoxicity, and complete biodegradability make them environmentally friendly agents that align with the principles of organic and sustainable agriculture [4,5].

The history of using phages for plant protection spans a century, dating back to the first documented case in 1924 [3]. Since then, a substantial body of evidence for their efficacy has accumulated, ranging from pre-sowing seed treatment to protecting vegetative plants and extending crop storage life. Successful examples include the suppression of bacterial rice blight, kiwifruit canker, and solanaceous wilt [4,6,7]. In response to modern demands for agricultural environmental safety, a whole class of industrial bioproducts based on bacteriophages has emerged on the market. These utilize not only individual phages but also so-called “phage cocktails,” which contain two, three, or even six lytic phages [8]. Phage preparations are widely used in animal husbandry [9,10,11,12], whereas only a few such preparations are known for crop production [13].

However, the efficacy of these agents directly depends on the diversity and specificity of the phage communities themselves, which are highly sensitive to anthropogenic impact. Despite the ubiquitous presence of phages, their composition and functional potential in agroecosystems (agrocenoses) are subject to significant influence from environmental factors and farming practices. Metagenomic studies indicate, for example, the accumulation of antibiotic resistance genes in the soil virome [4,14,15]. A particularly critical factor is the farming system: it has been proven that a shift to monoculture leads to a sharp depletion of phage communities, and their diversity varies significantly depending on the type of ecosystem and land use intensity [16,17]. Thus, the search for and study of new bacteriophages adapted to specific agrocenoses is a pressing task for developing innovative means of managing plant “health”.

Leguminous crops (alfalfa, soybean, lentils, peas) play a key role in sustainable agriculture by enhancing soil fertility through symbiotic nitrogen fixation via nodule bacteria (rhizobia). However, rhizobia themselves are susceptible to infection by specialized bacteriophages, known as rhizobiophages [18]. Historically, rhizobiophages have been successfully used for phage typing and identification of rhizobial strains [19,20,21]. However, phages are key regulators of rhizobial abundance in soil: by reducing bacterial density in the rhizosphere, they can negatively impact nodulation processes and, consequently, the efficiency of nitrogen fixation [18,22].

This issue is of particular practical significance in the context of applying bacterial inoculants during sowing. The titer of the introduced highly effective inoculant strain can be significantly reduced due to the lytic activity of resident soil phages [23,24]. Such microbe–phage interactions occur at all stages of plant development—from seed germination to the vegetative stage.

Therefore, ensuring the efficacy of bioproducts comes with two challenges: the first is finding ways to enhance the phage resistance of inoculant strains; the other is conducting fundamental research into the soil virome of agrocenoses, including the composition and properties of autochthonous phages. However, current knowledge about the species diversity of phages in agrocenoses and their biological characteristics remains extremely limited.

Currently, 201 phage genomes specific to rhizobia (root nodule bacteria) have been deposited in the NCBI database. The vast majority of these (71%) are specific to representatives of the genus *Rhizobium*, among which 109 are genomes of phages specific to bean symbionts (*R. phaseoli*, *R. etli*). Meanwhile, the share of phages specific to bacteria of the genus *Sinorhizobium* does not exceed 13% (27 phages), with 17 phages of *S. meliloti*, symbionts of alfalfa, sweet clover, and fenugreek, being the predominantly known ones.

Most known rhizobiophages (84%, or 169 out of 201) belong to the realm *Duplodnaviria*, which comprises double-stranded DNA viruses encoding a major capsid protein with the HK97 fold. Small viruses predominate in terms of genome size: 145 isolates have a genome of less than 100 kbp, 46 phages range from 100 to 200 kbp, and only nine isolates possess genomes exceeding 200 kbp. Recently, we described the first giant (jumbo) sinorhizobiophage, AP-J-162, with a record-breaking genome for this group, sized at 471.5 kbp and encoding 711 predicted genes, with more than half (55%) of their protein products being unique [25]. Bacteriophage genomes are increasingly recognized as mosaic entities, with horizontal gene transfer (HGT) shaping their evolution across distantly related bacterial phyla [26]. Nevertheless, cross-phylum HGT has not been documented in rhizobiophages.

Rhizobiophages exhibit various morphotypes, including siphoviruses, podoviruses, and myoviruses. Representatives of the family *Microviridae* (realm *Monodnaviria*) [27], phages with filamentous morphology [28], and viruses whose taxonomic position is not defined even at the realm level, have also been described. Notably, according to modern taxonomy, almost half (49%) of all known rhizobiophages belong to unclassified taxa [29]; even such model objects as *Sinorhizobium* phage phiLM21 and *Rhizobium* (*Sinorhizobium*) phage 16-3 fall into this category [30,31].

Despite growing interest in legume phages in recent years, knowledge of the genomic diversity and, most importantly, biological activity and ecological impact of rhizobiophages—particularly viruses infecting symbionts of the highly valuable forage crop alfalfa—remains extremely fragmented and unsystematized. Rhizobial populations are known to comprise two subpopulations: soil strains, which are highly competitive but typically low-effective, and bacteria released from nodules, which are highly effective [32,33]. Using phage typing, Bromfield et al. [34] showed that phage-resistant and phage-susceptible isolates from alfalfa and sweet clover nodules represented distinct genospecies and even different genera, with resistant strains often exhibiting reduced symbiotic effectiveness. Thus, phage susceptibility patterns can serve as a marker for ecologically distinct subpopulations. However, no data are available on rhizobiophage behavior toward these two subpopulations, and the role of rhizobiophages as a selective filter regulating rhizobial populations and threatening inoculant efficacy remains severely understudied. Critically, their role as active regulators of rhizobial populations and a potential threat to the efficacy of bacterial inoculants in agroecosystems remains severely understudied. In particular, no information is available on how rhizobiophages interact with distinct natural subpopulations of *S. meliloti*—specifically, soil versus nodule-derived strains, and isolates from phytocenoses versus agrocenoses.

The presence of virulent, autochthonous phages in agricultural soils poses a direct risk to applied microbial bioproducts (inoculants). Upon introduction, highly effective commercial inoculant strains are vulnerable to predation by native phage assemblages, leading to their rapid depletion and a significant reduction in viable titer. This, in turn, can compromise nodulation, nitrogen fixation, and ultimately crop yield [24]. Hence, to evaluate the real threat to inoculants and develop effective countermeasures, a detailed understanding of key phenotypic properties—such as host range, infection kinetics, environmental stability, and soil prevalence—is essential.

This work presents a comprehensive characterization of a new lytic rhizobiophage, AP-20-A, isolated from agricultural soil. Although phylogenomic analysis places AP-20-A only distantly within the *Schitoviridae* family, the phage carries multiple genes with closest homology to *Paenibacillus* bacteria and their phages, indicating cross-phylum HGT between Gram-positive (*Bacillota*) and Gram-negative (*Alphaproteobacteria*) lineages, a phenomenon not previously documented in *Sinorhizobium*-infecting phages. Emphasizing its biological properties alongside genomic analysis, we have determined its host range and infection parameters against a collection of native *Sinorhizobium meliloti* strains from various geographical zones. Furthermore, the study identified an *S. meliloti* strain resistant to AP-20-A infection, providing a first step towards understanding phage-resistance mechanisms and designing protective strategies for inoculants.

## 2. Results

Phage AP-20-A was isolated from a soil sample from an agricultural field in the Voronezh region using an enrichment technique (see Section 4; [35,36]).

### 2.1. Morphological Characteristics of the Phage AP-20-A

**Figure 1:**
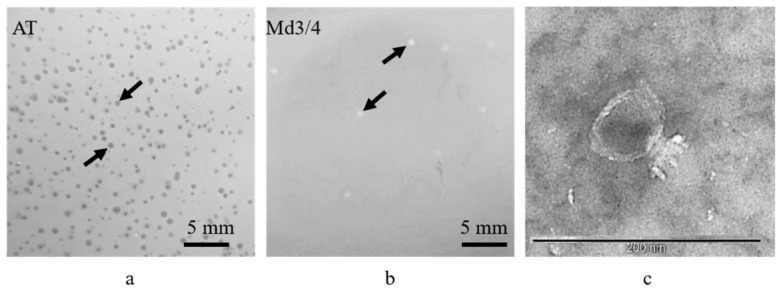
Plaques and transmission electron microscopy (TEM) of the rhizobiophage AP-20-A. (**a**,**b**) Plaques (indicated by arrows) formed by the phage on lawns of *S. meliloti* strains AT (**a**) and Md3/4 (**b**) using 0.4% semi-solid agar. (**c**) TEM micrograph of phage AP-20-A particles.

Phage AP-20-A formed small, transparent plaques (up to 1 mm in diameter) on an *S. meliloti* AT lawn using 0.4% agar plates after 32 h of incubation (Figure 1a). In contrast, on an *S. meliloti* Md3/4 lawn, it produced weak, opaque plaques surrounded by a lysis zone of up to 2 mm in diameter under the same conditions (Figure 1b).

Transmission electron microscopy (TEM) revealed that phage AP-20-A virions had an icosahedral head (60 ± 5 nm in length and 80 ± 5 nm in width) and a short, non-contractile tail (20 ± 5 nm in length) with fibers (Figure 1c). Based on this morphology, phage AP-20-A has a podovirus morphotype.

A comparative analysis of virion size was performed for phage AP-20-A, canonical podoviruses listed by the ICTV (https://ictv.global/report_9th/dsDNA/Podoviridae (accessed on 6 December 2025)), and three known *Sinorhizobium* podoviruses (phiM5, phiM6, ort11). The typical capsid diameter for representatives of different families—such as *Uetakevirus* (*Uetakevirus* epsilon15), *Bruynoghevirus* (*Bruynoghevirus* LUZ24), *Schitoviridae* (*Enquatrovirus* N4), and order *Autographivirales* (*Teseptimavirus* T7)—ranges from 60 to 70 nm, with a short non-contractile tail of up to 20 nm in length with fibers. The capsid size of AP-20-A is smaller than that of phage phiM5 (75 nm [37]) but larger than that of phage ort11 (54 nm [28]).

Thus, phage AP-20-A shares morphological characteristics common to podoviruses. In terms of virion size, it is similar to both typical and known podoviruses infecting *Sinorhizobium* spp.

### 2.2. Host Range and Efficiency of Plating (EOP) of Phage AP-20-A

*Spot test*. The lytic activity of phage AP-20-A was assessed using a spot test assay on solid agar medium. The host range panel consisted of 78 randomly sampled native isolates and four highly efficient (Eff^++^) strains of *Sinorhizobium meliloti.* Additionally, five strains of *S. medicae* (a closely related species), the strain *S. fredii* DSM5851 from the same family, nine non-rhizobial strains (including four species of *Bacilli*) were tested for phage specificity (see Section 4). Phage AP-20-A demonstrated lytic activity against 43 out of 78 native strains of *S. meliloti*, against three Eff^++^ strains of *S. meliloti* (46 out of all 82 *S. meliloti* strains tested in total), and against two strains of *S. medicae*, but not against *S. fredii* DSM5851. In total, 35 native and one Eff^++^ strain *S. meliloti* were resistant to the phage. Furthermore, the phage showed no lytic activity against any of the tested non-*Sinorhizobium* bacterial species (see Section 4), confirming its narrow host specificity. Consequently, the host range breadth of phage AP-20-A against its primary host, *S. meliloti*, was calculated to be 0.56 (95% CI: 0.45–0.67; confidence interval for the proportion was calculated using the Clopper–Pearson method, see Section 4).

*EOP analysis*. To identify differences in infection efficiency against various *S. meliloti* strains, the efficiency of plating (EOP) was assessed for the 43 phage-sensitive *S. meliloti* strains, including *S. meliloti* strain AT. The latter was chosen as the reference strain for EOP analysis because it was completely lysed by the phage (EOP = 1) and served as the laboratory host.

Analysis of phage activity showed that high (EOP ≥ 0.5) and medium productive infection (0.1 ≤ EOP < 0.5) were observed for 7% and 19% of the 43 studied *S. meliloti* strains, respectively. Low productive infection (0.001 < EOP < 0.1) and inefficient infection (EOP ≤ 0.001) were observed for 14% and 60% of the 43 strains tested (6 and 26 strains, respectively). Regarding the three Eff^++^ strains, low productive and inefficient infection were observed for all three strains.

Thus, *S. meliloti* strains cluster into four EOP groups based on their sensitivity to phage AP-20-A. The vast majority (26 out of 43) of these strains (frequency 0.60) support inefficient infection (EOP ≤ 0.001), which may be consistent with putative pseudolysogeny.

### 2.3. Ecological Specificity of Phage AP-20-A

**Figure 2:**
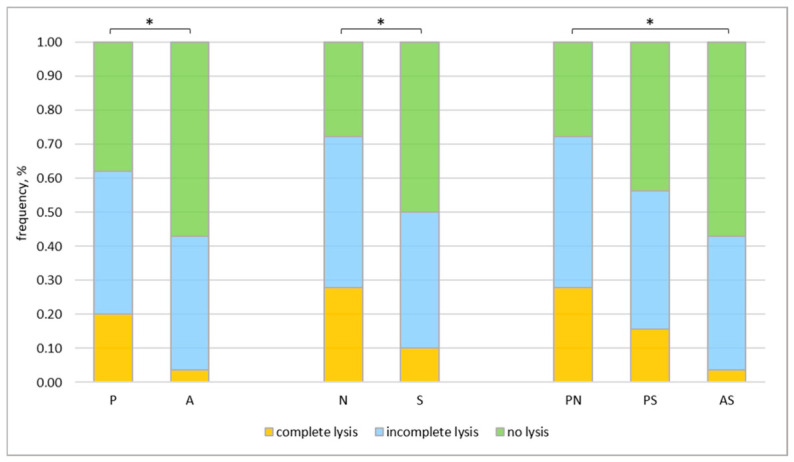
Lysis frequency of phage AP-20-A against *S. meliloti* strains from phytocenoses (P) and agrocenoses (A), as well as against the “nodule” (N) and “soil” (S) subpopulations. Designation: PN, phytocenoses “nodule” subpopulation; PS, phytocenoses “soil” subpopulation; AS, agrocenoses “soil” subpopulation; statistically significant differences are marked with an asterisk (*).

For the ecological specificity analysis aimed at identifying differences in susceptibility to phage AP-20-A between the defined subpopulations (see Section 4, Figure 2), only strains showing “no lysis” (35 strains) and “complete lysis” (11 strains) were selected based on the spot test results (see Section 2.2). The proportion of strains showing “incomplete lysis” was similar across all compared subpopulations, with an average frequency of 0.41 ± 0.02. Therefore, this group of isolates was excluded from further analysis without introducing systematic bias.

Strains from phytocenoses were more susceptible to the phage than strains from agrocenoses, with lysis frequencies of 0.20 and 0.04, respectively (10 out of 50 and 1 out of 28; Fisher’s exact test, P_F_ = 0.036). No significant differences in phage susceptibility were detected between the soil subpopulations from phytocenoses and agrocenoses (P_F_ > 0.05). In contrast, nodule strains from phytocenoses (PN) were significantly more phage-sensitive than soil strains from agrocenoses (AS), with lysis frequencies of 0.28 (5 out of 18) and 0.04 (1 out of 28), respectively (P_F_ = 0.015). Furthermore, the occurrence of phage-resistant strains was higher among soil isolates than among nodule strains (P_F_ = 0.043).

The obtained data suggest that the phage selectively lyses a specific subpopulation of the host bacterium—namely, strains released into the soil upon the natural senescence of root nodules. It was also demonstrated that in agrocenoses, a substantial proportion of local strains exhibit resistance to the phage derived from an analogous microbiota. These results demonstrate a potential ecological filter: while the phage AP-20-A is active in both environments, agrocenoses harbor a substantially higher natural prevalence of phage-resistant *S. meliloti* strains compared to phytocenoses. This native resistance underscores the potential vulnerability of non-adapted, introduced inoculant strains that may lack such defenses in the same soil.

### 2.4. Phage–Microbe Interactions

**Figure 3:**
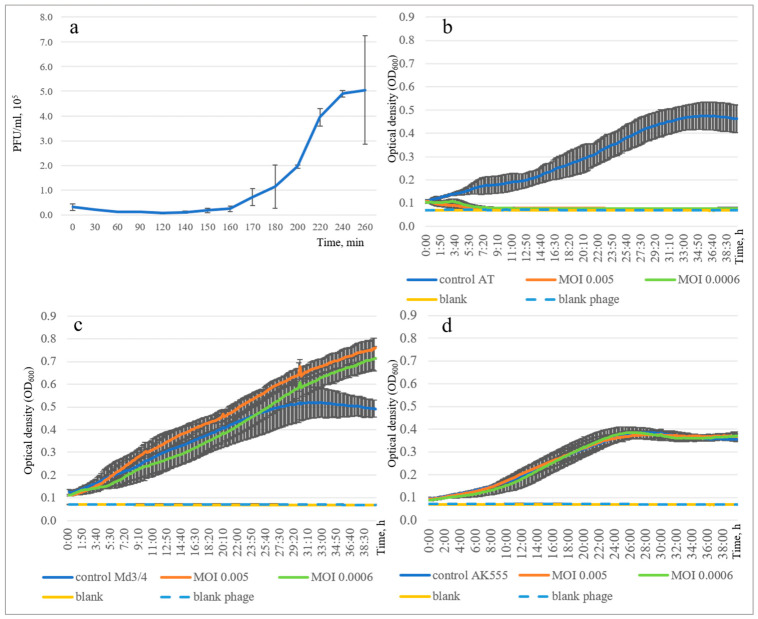
Replication kinetics and infection dynamics of *Sinorhizobium* phage AP-20-A. (**a**) One-step growth curve of phage AP-20-A. (**b**–**d**) Phage AP-20-A infection curves of differentially resistant *S. meliloti* strains. (**b**) *S. meliloti* strain AT, (**c**) strain Md3/4, (**d**) strain AK555. Designations: blue line, growth curve of the uninfected strain (control); orange and green lines, growth curves of the strain infected with the phage culture, respectively, at MOI 0.005 and 0.0006; blank, optical density values of the medium in which the cultivation was carried out; blank phage, optical density values of the phage lysate sample without the addition of bacterial culture.

To evaluate the phage’s lytic efficacy and potential alternative infection outcomes, we first analyzed the key reproductive parameters of phage AP-20-A using a one-step growth curve on the sensitive *S. meliloti* AT strain. AP-20-A exhibited a latent period of 160 min and an average burst size of 27 PFU/cell (Figure 3a). Notably, these kinetics differ significantly from those of the podovirus sinorhizobiophage ort11, which has a shorter latent period (90 min) and a smaller burst size (19–20 PFU/cell) [28]. The duration of the latent period is known to be controlled by phage-encoded proteins, particularly holins, and depends on bacteria cell metabolism [38]. We hypothesize that the extended latent period of AP-20-A, coupled with its higher burst size, reflects fundamental differences in its lysis-lysogeny system, holin-endolysin timing, and virion assembly pathways compared to the model sinorhizobiophage ort11.

To assess how these reproductive parameters translate into population-level outcomes, the lytic efficacy of AP-20-A was then tested against three *S. meliloti* strains representing different susceptibility profiles in liquid medium. The study included two phage-sensitive strains (AT and Md3/4) and one phage-resistant Eff^++^ strain (AK555), as pre-determined by spot assay. Infections were carried out at two multiplicity of infection (MOI) values: 0.0006 and 0.005. Bacterial growth and lysis were monitored continuously; data from specific timepoints—0 h (infection point), 1.5 h, 4.5 h, 7.0 h (onset of stationary phase), and late stationary phase (≥24 h)—were selected for detailed analysis.

#### 2.4.1. Efficient Lysis of a Fully Sensitive Host (Strain AT)

Infection of the phage-sensitive *S. meliloti* strain AT with the phage at MOI 0.0006 and 0.005 resulted in a rapid decrease in the number of viable cells (Figure 3b, Table S1). Within just 7 h, the bacterial titer decreased by approximately 50% compared to the control (uninfected) culture (OD_600_ infected 0.086 ± 0.008 and 0.080 ± 0.003 for MOI 0.0006 and 0.005; control 0.163 ± 0.024; n = 4). By this time, viability was maintained in no more than 28% of the cells compared to the initial infected culture (initial OD_600_ = 0.108 ± 0.008; n = 4). The difference in lysis efficiency between the two MOI values was negligible.

In contrast to the control culture, which reached the stationary growth phase by 33 h (OD_600_ = 0.459 ± 0.053; n = 4), the infected cultures showed no signs of regrowth for up to 40 h, indicating complete and irreversible lysis of the AT strain culture. The reduction in titer of the infected AT strain culture is consistent with the results of the spot test and the double agar overlay method, which recorded complete clearance of the bacterial lawn with the formation of clear plaques (Figure 1a).

Thus, bacteriophage AP-20-A effectively lyses the *S. meliloti* AT culture, leading to complete bacterial cell death without regrowth.

This rapid and complete population collapse is consistent with the phage’s reproductive parameters. Given its latent period of ~160 min, the observed lysis within 7 h corresponds to approximately 2–3 consecutive infection cycles, each producing a substantial burst of ~27 new viral particles per lysed cell, corresponding to approximately 2.84 × 10^6^ and 3.54 × 10^5^ generated PFU for MOIs of 0.005 and 0.0006, respectively. Together, the high burst size and short latent period explain the potent lytic activity of AP-20-A against susceptible rhizobial strains.

#### 2.4.2. Putative Lysogenic Response (Strain Md3/4)

The second tested phage-sensitive strain, selected based on spot test results, was *S. meliloti* Md3/4. However, unlike strain AT, infection with phage AP-20-A did not result in lysis but induced complex alterations in the bacterial growth dynamics (Figure 3c, Table S1).

The initial infection phase (0–7 h) was characterized by moderate suppression. In the first hours post-infection, a minor reduction in the optical density of the culture—up to 12.6% compared to the control—was observed (the differences were not statistically significant at *p* < 0.05). By 7 h, suppression persisted at MOI 0.0006 (15.9%), whereas at MOI 0.005, the optical density of the infected culture had already returned to the control level (0.208 ± 0.026 and 0.201 ± 0.056 for the infected and control cultures, respectively; n = 4).

By the time the control culture reached the stationary phase (27.5 h, OD_600_ = 0.492 ± 0.096), the infected cultures not only reached these density values but exceeded them by 4.5% at MOI 0.0006 and 19.7% at MOI 0.005 (OD_600_ = 0.514 ± 0.043 and 0.589 ± 0.027, respectively; n = 4).

By the 40-h mark of cultivation, a sustained stimulatory effect on bacterial growth was observed. The infected culture continued its exponential growth phase, with its density exceeding the final control level by an average factor of 1.45 times (as the control culture had ceased growing). Compared to the initial values (OD_600_ = 0.113 ± 0.004 and 0.111 ± 0.003 for MOI 0.0006 and 0.005, respectively; n = 4), the density of the infected culture had increased by a factor of 6.2 to 6.7.

Therefore, infection of the Md3/4 strain with phage AP-20-A did not suppress bacterial growth; instead, it caused an initial delay followed by significant stimulation. This pattern, coupled with the formation of turbid plaques (Figure 1b)—a hallmark often associated with temperate phages [39]—is consistent with a putative lysogenic interaction. However, we note that definitive confirmation of lysogeny (e.g., detection of integrated prophage or stable phage carriage as chronic infection) was not performed, and alternative explanations such as pseudolysogeny or metabolic adaptation cannot be ruled out. The observed delay of ~7 h aligns with the duration of a full lytic cycle for AP-20-A (latent period ~160 min), potentially suggesting that this interval is critical for determining the infection outcome (lysis vs. lysogeny) in this host strain. Considering the decrease in bacterial culture density during the lytic cycle, the numbers of phage particles produced were 6.82 × 10^5^ and 8.50 × 10^4^ PFU for MOI 0.005 and 0.0006, respectively.

#### 2.4.3. Absence of Lytic Activity Against a Resistant Host (Strain AK555)

Infection of the phage-resistant strain AK555 with phage AP-20-A revealed no significant difference in growth compared to the uninfected control. All cultures, including the control, entered the stationary phase by 24 h, attaining similar optical densities (0.350 ± 0.019 for the control, 0.363 ± 0.024 at MOI 0.0006, and 0.352 ± 0.037 at MOI 0.005; *p* > 0.05; n = 4) (Figure 3d, Table S1). These findings demonstrate that phage AP-20-A is not lytic for strain AK555 and there was no production of new phage particles. The potential for a lysogenic interaction, however, cannot be excluded and requires further study.

Thus, the lytic activity of AP-20-A against *S. meliloti* strains with varying phage susceptibility highlights the host-specific nature of the phage–microbe interaction. This spectrum of host-specific interactions—from rapid lysis (strain AT) to potential lysogeny (Md3/4) and complete resistance (the Eff^++^ strain AK555)—prompted us to perform a genomic analysis of phage AP-20-A to identify the genetic determinants underlying its infectivity and host range (detailed below).

#### 2.4.4. Putative Defense Systems in *S. meliloti* Md3/4 and AK555

To explore the genetic basis of differential susceptibility to phage AP-20-A, anti-phage defense systems were predicted using PADLOC and CRISPR/Cas Finder. Both strains carried a highly similar type II restriction-modification system (Identity = 99.9%; Coverage = 100%). Strain AK555 (resistant) contained an additional unique AbiE abortive infection system compared to strain Md3/4 (sensitive), while Md3/4 carried more predicted CRISPR-Cas cassettes (six vs. one in AK555). Spacers from Md3/4 CRISPR cassettes showed homology to other rhizobiophages, but not to AP-20-A. These observations suggest that the additional AbiE system may contribute to resistance, whereas the conserved R-M system and CRISPR-Cas arrays are unlikely to be primary determinants of resistance to AP-20-A. Experimental validation would be required to establish a causal link.

### 2.5. Phage AP-20-A Genome

The genome of phage AP-20-A was sequenced, assembled, and annotated. The genome sequence has been deposited in GenBank under the accession number PX794722.

#### 2.5.1. Nucleotide Sequence Analysis of AP-20-A

**Figure 4:**
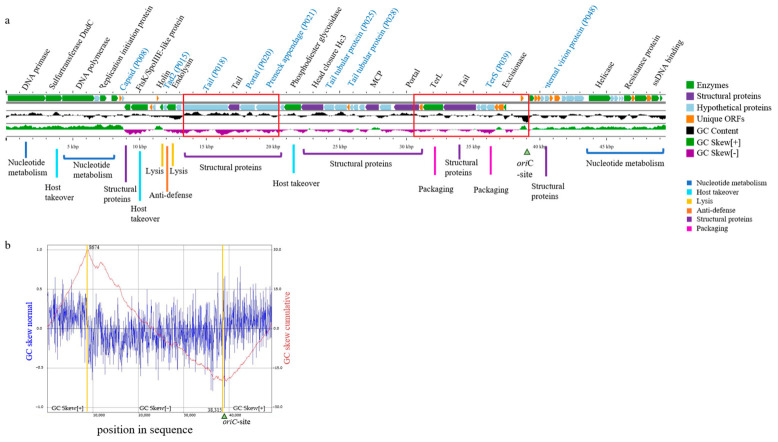
Physical map of the AP-20-A phage genome. (**a**) Distribution of CDSs in the linear genome of AP-20-A relative to each other and to regions with different GC content (see Section 2.5.2). Regions of likely horizontal gene transfer (HGT) events are circled in red. (**b**) GC skew plot (Step Size: 50, Window Size: 100). The blue line represents the normal GC skew graph (y axis on the left). The red line represents the cumulative GC skew graph (y axis on the right). The yellow line indicates the maximum (coordinate 8674) and minimum (coordinate 38,315) of the cumulative GC skew graph. The region with higher-than-average GC content is labeled as GC Skew[−]; the region with lower-than-average GC content is labeled as GC Skew[+] (see text). 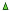 indicates the putative *oriC* site at coordinates 38.7–39.2 kb (see text).

The genome of phage AP-20-A is a double-stranded linear DNA molecule of 49,426 bp in length with an average GC content of 43.19% (Table 1; Figure 4). Nucleotide sequence analysis revealed variations in the GC composition of AP-20-A, namely extended regions with a higher (43.69%; GC Skew[−] coordinates 8.7–38.3 kb) or lower (42.44%; GC Skew[+] coordinates 38.3–49.4 kbp and 0–8.7 kbp) GC content compared to the average (Table 1, Figure 4a,b). The GC Skew[−] region was 1.5 times larger than the GC Skew[+] region (29.9 kbp and 19.5 kbp, respectively (Figure 4a,b). The putative origin of replication (*oriC*) was located close to the minimum of the GC skew cumulative graph (Figure 4b).

The nucleotide sequence of phage AP-20-A is unique. Comparative genomic analysis revealed no significant similarity between the AP-20-A genome and a set of 21 rhizobiophage genomes belonging to different families and infecting various host bacterial species (see Section 4; Table S6). The Mauve aligner detected no significant genomic synteny (i.e., less than 60 bp of locally colinear blocks) with three *Sinorhizobium*-infecting phages (phiM5, phiM6, and ort11), 17 *Rhizobium* phages, or the *Mesorhizobium* phage vB_MloP_Lo5R7ANS (Figure S1). However, five and six genome fragments of up to 450 bp were found to be similar to the genomes of phages SV21 and vB_PlaP_API480, respectively, which infect bacteria of the genus *Paenibacillus* (see Section 4; Table S6). The average identity values of these fragments were 64.6% and 65.17%, respectively, with 2% coverage according to BLASTn. These fragmentary similarities to phages of *Paenibacillus* (a genus taxonomically distant from *Sinorhizobium*) are suggestive of horizontal gene transfer (HGT) between different bacterial phyla—specifically, between *Bacillota* and *Alphaproteobacteria*.

To further predict functional acquisitions via HGT, two regions of likely horizontal gene transfer were identified using Alien Hunter, spanning 7.2 and 8.6 kbp (coordinates 13,286–20,439 and 30,610–39,196, respectively; Figure 4a). The first HGT region (13,286–20,439) was predicted to encode structural proteins: tail proteins (p018 and p019), portal protein (p020), and pre-neck appendage protein (p021) (Table S3). Thus, this interphylum transfer likely contributed to phage morphogenesis. The second HGT region (30,610–39,196) contained CDSs predicted to encode two terminase subunits (p035 and p039), excisionase (p042), tail protein (p036), as well as fragments of portal protein (p033) and the *oriC* region. This region is therefore predicted to be involved in DNA packaging and replication regulation. The presence of these two large HGT regions, combined with the *Paenibacillus* phage similarities, suggest that phage AP-20-A has undergone multiple interphylum HGT events, acquiring functionally important modules from distantly related phages. As with the complete genome sequence, no significant similarity was found for these regions using BLASTn against rhizobiophages, which is consistent with exogenous origin.

Key non-coding regulatory elements were identified in the AP-20-A genome. A putative origin of replication (*oriC*) was found at position 38,786–39,227, representing a single, compact locus spanning 442 bp. This architecture contrasts with that of the well-studied sinorhizobiophage ort11, which possesses two distinct *oriC* regions (123 bp and 543 bp), highlighting a fundamental divergence in their replication initiation systems.

A search for transfer RNA (tRNA) genes using tRNAscan-SE revealed that the AP-20-A genome does not encode any tRNAs. This obligate dependence on the host’s translational machinery provides crucial context for the subsequent codon usage analysis.

A comparative analysis of codon usage frequency was performed for phage AP-20-A and its bacterial host, *S. meliloti*. The phage employs the universal genetic code. A significant divergence in codon preference was revealed: for 80% of codons, the difference in usage frequency between the phage and bacterium ranged from 0.12 to 0.52. A key finding was the phage’s active employment of 12 codons that are rare in the host genome (e.g., Ala-GCT, Ile-ATA, Arg-AGA/AGG), with a mean frequency of 0.27 compared to 0.03 in the bacterium.

Codons for five aliphatic amino acids (Lys, Leu, Gly, Glu, Ala) were most abundant in the phage genome. This correlates with the presence of multiple copies of the corresponding tRNA genes in the host’s genome. Similar usage frequencies between the phage and host were observed only for the codons Tyr, Ser(AGC), Arg(CGG, CGA), and the stop codon TAG.

Thus, phage AP-20-A exhibits a dual strategy in codon usage. It follows a typical phage adaptation by preferentially using host-abundant codons corresponding to multiple-copy tRNA genes (Lys, Leu, Gly, Glu, Ala). Concurrently, it actively utilizes a set of 12 host-rare codons. This latter feature may represent an adaptation to modulate translation efficiency or reduce dependence on the host’s tRNA pool, potentially allowing for finer control over its own protein synthesis rates, according to [40].

Lifestyle prediction for phage AP-20-A, performed using the automated PhageScope v1.3 pipeline and the machine learning-based PhageGE web server, consistently indicated a virulent/lytic nature. AP-20-A was classified as virulent by PhageScope and as lytic with 93.0% probability by PhageGE.

Overall, the exceptionally low genomic similarity to known rhizobiophages, the distinct organization of its replication origin, the lack of tRNA genes, and its unique codon usage profile, and the predicted strictly lytic lifestyle collectively confirm that phage AP-20-A does not belong to common *Sinorhizobium* phage lineages. It represents a novel and divergent genomic group. Importantly, the identification of interphylum HGT regions (with similarity to *Paenibacillus* phages) and the functional prediction of acquired modules (structural proteins, terminases, excisionase) provide specific evidence for cross-phylum gene transfer shaping the functional repertoire of this phage. This profound nucleotide-level uniqueness warranted a detailed investigation of its protein-coding potential, presented in the following sections.

#### 2.5.2. Functional Annotation of AP-20-A CDS

Analysis of the AP-20-A genome identified 66 protein-coding sequences (CDSs), while no CDSs encoding phage-specific tRNAs were detected (Table 1). Functions were predicted for only 24 of the 66 CDS products, with the vast majority encoding hypothetical proteins, as confirmed by BLASTp analysis (Table 1). Of these, five CDSs encoded virion structural components, and 19 encoded enzymes belonging to various functional groups (Table S2).

##### Phage Particle Morphogenesis and Packaging Module

Five CDSs—p019, p024, p031, p033, and p036—were identified as encoding structural components of the virion (Table 2).

The amino acid sequence of the P019 protein, a phage tail tape measure protein, showed similarity to a corresponding protein identified in the *Donellivirus* gee prophage (*Bacillus* phage G; Identity = 67.20%; Coverage = 43%) within the genome of *Priestia aryabhattai*, a plant growth-promoting rhizobacterium (PGPR) associated with alfalfa [41]. Notably, structural modeling of P019 using the I-TASSER web server revealed its closest structural analog to be the antimicrobial peptidase lysostaphin from *Staphylococcus simulans* (Identity = 20.3%; Coverage = 82.0%). The model also identified structural similarities to proteins from other phylogenetically distant bacteria and plants in metagenomic databases. This intriguing combination of sequence homology to a prophage within an alfalfa-associated PGPR and structural resemblance to a bacteriolytic enzyme suggests that P019 may have evolved specialized functions. Beyond its structural role in the virion, P019 could potentially facilitate infection by interacting with or degrading components of the bacterial cell wall. This molecular adaptation might underlie the phage’s specificity and efficacy against bacterial hosts within the alfalfa rhizosphere, a niche it shares with PGPR.

The amino acid sequences of the portal protein P033 and the major capsid protein P031 showed the highest coverage with proteins encoded by CDS of the *Bacillus* phage BM5 and *Donellivirus* gee prophages within the genomes of *Paenibacillus larvae* and *Psychrobacillus* sp. (Coverage = 89% and 94%, respectively; Identity up to 53.31%, Table 2). According to the I-TASSER Web Server, the closest structural analogs for proteins P033 and P031 were the capsid asymmetric unit of cyanophage Pam1 and the Portal Protein of *Lederbergvirus* P22 (*Salmonella* virus P22), respectively. A high degree of coverage was also observed between the amino acid sequence of the head closure protein P024 and a protein from the podovirus *Paenibacillus* phage vB_PlaP_API480 (Identity = 36.66%; Coverage = 91%). The closest structural analog for P024 was a head-to-tail adapter from podovirus infecting *Paenibacillus*. These results consistently demonstrate that the core structural proteins of phage AP-20-A share the highest homology—both in sequence and structure—with those of podoviruses infecting *Bacillus* and related *Bacillota* (*Paenibacillus*, *Psychrobacillus*), rather than with known rhizobiophages. This pattern strongly suggests an evolutionary origin of its morphogenetic module distinct from typical sinorhizobiophages, likely acquired through horizontal gene transfer from phages targeting phylogenetically distant soil bacteria.

The amino acid sequence of the tail fiber protein P036 exhibited similarity to the corresponding protein in *Pagevirus* palmer (Identity = 39.60%; Coverage = 18%; Table 2). The closest structural analog for P036, identified by the I-TASSER Web Server, was an arabinanase from the thermophilic bacterium *Geobacillus stearothermophilus*, an organism ubiquitous in soil, hot springs, and ocean sediments, and known for its role in food spoilage [42]. The structural homology of tail fiber protein P036 to a bacterial arabinanase points to a specialized mechanism for host recognition. This adaptation likely enables AP-20-A to target specific polysaccharide structures on the surface of rhizobial cells in the soil environment.

The genome packaging module of AP-20-A includes p035, which encodes the terminase large subunit (COG category X, PHROG_2), a key enzyme responsible for viral DNA packaging. The closest homolog of this protein is from a *Paenibacillus* phage vB_PlaP_API480 (Identity = 47.06%; Coverage = 98%, Table 2). Notably, no significant similarity was detected with the corresponding proteins from the rhizobiophages ort11 and phiM5 (Coverage < 5%). This stark contrast underscores the significant divergence in the DNA packaging system of AP-20-A compared to model rhizobiophages.

Significant interest also lies in the p011 gene of phage AP-20-A. Its predicted product is a protein of the FtsK/SpoIIIE family (COG category D, PHROG_1358), whose functions are not fully understood but are predicted to involve DNA binding [43]. The P011 protein of phage AP-20-A is similar to an FtsK/SpoIIIE family protein from *Caudoviricetes* sp. (Identity = 35.37%; Coverage = 92%, Table 2). FtsK/SpoIIIE family proteins are ATP-dependent DNA translocases. This has led to the hypothesis that they may participate in the phage genomic replication complex during the “rolling circle” mechanism, facilitating the disentanglement of long concatemeric DNA chains [43]. Furthermore, the P011 protein might be involved in resolving concatemeric DNA during its packaging into the capsid [44]. The proposed function of P011 is consistent with the presence of the terminase large subunit gene (p035) in the AP-20-A genome. A phage possessing such a protein may gain a selective advantage by achieving significantly enhanced speed and fidelity in viral DNA packaging compared to phages lacking such a terminase accessory protein [45,46].

Collectively, these results demonstrate that the core structural proteins of phage AP-20-A, including those for capsid assembly, DNA packaging, and host recognition, share their closest evolutionary relationships with podoviruses infecting bacteria of the phylum *Bacillota* (genera *Bacillus*, *Paenibacillus*, *Psychrobacillus*), rather than with known sinorhizobiophages.

##### Phage Replication and Transcription

Eight CDSs (p003, p001, p055, p005, p044, p062, p064, p065) were implicated in DNA replication, recombination, and repair (COG category L). These include a DNA polymerase I (P003), a key enzyme known for filling DNA gaps [47], as well, primase, nucleotide kinase, helicase, resolvase, Sak-like ssDNA annealing protein and ssDNA binding protein, Gp5.9-like inhibitor of RecBCD nuclease respectively (Table S2).

This functional group also belongs other two CDSs the p007 gene, which encodes a dNTPase (COG category F, PHROG_173) similar to dUTPase that found in *Bacillus* phage Staley (Identity = 41.29%; Coverage = 93%), and the p010 gene, a replication initiation protein (NOG373487, PHROG_2190) with a homolog identified in *Bacillus* sp. (Identity = 49.34%; Coverage = 99%).

Proteins of this family were first described in the temperate *Streptococcus* phage phiSfi21 but have since been identified in numerous phages infecting members of *Bacillota* (formerly *Firmicutes*) and *Pseudomonadota* (formerly *Proteobacteria*), as well as in the genomes of taxonomically diverse bacteria, including *Bacillota*, *Bacteroidota* (formerly *Bacteroidetes*), *Pseudomonadota*, *Fusobacteriota* (formerly *Fusobacteria*), and *Cyanobacteriota* (formerly *Cyanobacteria*) [48,49]. It has been demonstrated that bacteria encoding proteins of this family exhibit enhanced resistance to bacteriophages, although the underlying mechanism remains unclear [49].

Analysis of the spatial distribution of the identified CDSs showed a clear functional partitioning of the AP-20-A genome. Genes encoding replication, repair, and recombination proteins (including DNA polymerase, sulfotransferase, etc.) are predominantly located in the GC Skew[+] region, while genes responsible for virion morphogenesis and DNA packaging (structural proteins, terminase large subunit) are located in the GC Skew[−] region (Figure 4). Thus, the GC Skew[+] and GC Skew[−] regions differ not only in GC content but also in their functional assignment, supporting the modular architecture of the phage genome.

This functional partitioning is consistent with the predicted location of the replication origin (*oriC*). Downstream of *oriC*, genes involved in nucleic acid metabolism and host takeover are transcribed in the forward direction. Conversely, a block of genes oriented in the reverse direction, relative to *oriC*, encodes virion structural proteins and DNA packaging machinery. This canonical organization around the replication origin reinforces the observed modularity and likely reflects the coordinated spatiotemporal regulation of gene expression during the phage life cycle.

Summarizing the analysis of CDSs encoding phage particle morphogenesis, the packaging module, and phage replication and transcription, we conclude that AP-20-A combines several distinctive features: interphylum origin of structural genes in a lytic phage genome, structural homology of phage proteins to bacterial enzymes, and an FtsK/SpoIIIE family protein as part of the DNA packaging module. These characteristics distinguish AP-20-A from known podoviruses infecting rhizobia.

##### Predicted Host Takeover and Metabolic Modulation Functions

The genome of phage AP-20-A contains at least seven CDSs that may be involved in host takeover and metabolic modulation (Table S2).

*Lysis system.* Two CDSs (p014 and p016) are predicted to encode a holin (COG category X, PHROG_263) required for the release of mature virions, and an autolysin (hydrolase; COG category M, PHROG_435) that degrades bacterial cell wall peptidoglycan, respectively. Together, they may constitute a holin–autolysin system potentially involved in the timed release of progeny virions [50]. The predicted holin shows similarity to proteins from *Caudoviricetes* sp., while the putative autolysin is similar to a protein from *Macrococcus goetzii*.

*Putative cell surface adsorption protein*. Gene p023 is predicted to encode a protein belonging to the phosphodiester-glycosidase family (COG category G, PHROG_14029). Enzymes of this family cleave phosphodiester bonds in complex carbohydrates; it is therefore possible that this protein facilitates phage adsorption to the cell surface [51]. The predicted protein shares similarity with a sequence from the soil bacterium *Peribacillus frigoritolerans* (family *Bacillaceae*; Identity = 50.59%; Coverage = 99%), a strain known for its insecticidal properties and plant growth promotion [52].

*Predicted anti-defense candidates*. Three additional genes encode proteins that, by sequence analogy, may be involved in counteracting host defense systems:

- *p060* encodes a predicted Mu Gam-like end protection protein (PHROG_253; also annotated as a resistance protein in NOG244907). It shows similarity to the siphovirus Gp157 family protein (BLASTp, Table S2). One hypothesis is that this protein helps the phage evade bacterial immunity or provides superinfection exclusion against competing phages, but this interpretation is entirely based on *in silico* predictions.
- *p002* encodes a predicted sulfurtransferase DndC (COG category EH, PHROG_424). In bacteria, DndC is a component of the Dnd system involved in DNA phosphorothioation [53]. The phage-encoded homolog shows similarity to the enzyme from *Bacillus cereus* (Identity = 52%; Coverage = 94%). Whether this gene actually functions to counteract the host’s restriction-modification system remains unknown.
- *p026* encodes a predicted phosphodiesterase (COG category C, PHROG_19279), similar to a sequence from *B. pumilus* (Identity = 45.14%; Coverage = 100%). In some phages, phosphodiesterases have been reported to interfere with host antiviral systems or reprogram replication machinery [54]. Whether a similar role contributes to the lytic activity of AP-20-A is a testable hypothesis.

*Excisionase*. Gene p042 (66 aa) is bioinformatically predicted to encode an excisionase (COG category X, PHROG_2033), an enzyme typically involved in site-specific recombination during prophage excision. It shows sequence similarity to excisionases from *Phietavirus* phages infecting *Staphylococcus* species. Although AP-20-A is lytic in our assays, the presence of this gene may indicate either a historical lysogenic ancestor or a repurposed function in genome plasticity to lysogeny or pseudolysogeny.

In summary, the presence of a predicted holin–autolysin system, a putative phosphodiester-glycosidase, and several candidate anti-defense genes (DndC, phosphodiesterase, and the Mu Gam-like protein) suggests that AP-20-A may have the capacity to reprogram host cell functions and counter host immunity. The presence of multiple host-interaction genes with similarity to *Bacillota* homologs, rather than to *Alphaproteobacteria*, provides additional protein-level evidence for cross-phylum HGT. These bioinformatic predictions suggest directions for future research and already distinguish this phage from other known rhizobia-infecting podoviruses.

##### Analysis of Hypothetical Proteins

The majority of the predicted CDSs in phage AP-20-A remain functionally uncharacterized, encoding hypothetical proteins. Of the 66 predicted CDSs, 42 (63.6%) fall into this category, with an average length of 156.6 aa (range 29–1131 aa). Using SWISS-MODEL and InterProScan, 3D structural homology analysis was performed for all 42 CDSs, revealing that nine showed similarity to phage proteins, 20 to bacterial/eukaryotic/archaeal proteins, and 11 specifically to *Bacillota* proteins (Tables S3–S5). Only two CDSs (P050, P054) showed no detectable similarity to any known proteins.

*Phage-related CDSs*. Among the nine CDSs with similarity to phage proteins, six were assigned to putative structural components, including capsid-like proteins (P048, P008), a portal protein (P020), tail proteins (P028, P018, P025), and a neck protein (P021) (Figure 4). The latter, P021, is homologous to the pre-neck appendage protein of *Bacillus subtilis* podovirus φ29 (13.6% identity) and contains a pectin lyase-like domain (E = 3.0 × 10^−6^), suggesting a potential role in host cell wall degradation or adhesion. One CDS (P039) showed homology to a small terminase subunit.

**Figure 5:**
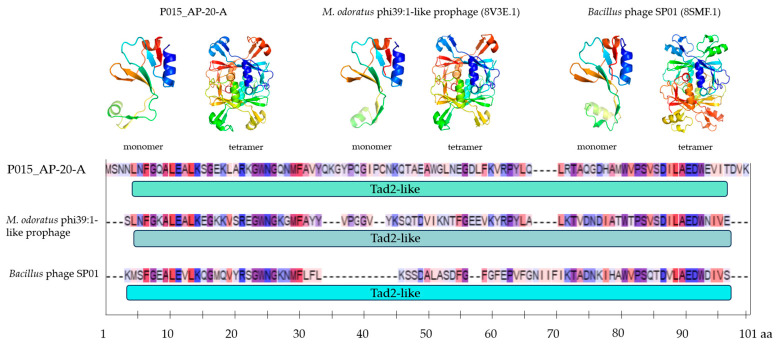
Structural modeling of the Tad2 protein P015 of *Sinorhizobium* phage AP-20-A. The upper part shows Richardson diagrams of Tad2 monomers and tetramers for AP-20-A, the phi39:1-like prophage of *M. odoratus*, and *Bacillus* phage SPO1. The lower part shows the amino acid sequence alignment of Tad2 proteins with mapped functional domains. The N-terminus is colored from blue to the C-terminus (red). Scale bars represent 5 amino acids (aa). Sequence alignment with standard “hydro” coloring for amino acid residues (hydrophobic groups).

*Tad2 protein encoded by gene p015*. According to InterProScan analysis, the product of gene p015 of phage AP-20-A contains a Tad2-like domain (PF11195). The first protein with this domain was described in *Bacillus* phage SP01; however, the greatest similarity of P015 was found to the MoTad2 protein (Identity = 50.54%; Coverage = 94%) from a prophage showing similarity to *Cellulophaga* phage phi39:1 in the genome of strain *Myroides odoratus* XSfan (RefSeq GCF_001549985.1). Similarity to Tad2 of phage SP01 is lower (Identity = 32.99%; Coverage = 97%) (Table S3, Figure 5).

Analysis of the amino acid sequences showed that the P015 protein of phage AP-20-A (97 aa, 10.9 kDa, pI 5.73) is longer than MoTad2 (88 aa, 9.9 kDa, pI 5.04) and Tad2 SP01 (89 aa, 10.03 kDa, pI 4.9) by 9 and 8 aa, respectively. Analysis of the Tad2-like domain (PF11195) in P015 revealed that it is also more extended (88 aa) than the domains of MoTad2 (83 aa) and Tad2 SP01 (79 aa). Functionally, MoTad2 inhibits the type II Thoeris system by binding His-ADPR, whereas Tad2 SP01 inhibits the type I Thoeris system by binding gcADPR [55]; however, both proteins belong to the same Tad2-like family (PF11195) and may perform a similar anti-defense function, despite their different targets.

The presence of a gene encoding a Tad2 protein in a rhizobiophage genome has not been reported previously. This makes P015 a candidate for further investigation, although its functional role remains to be determined.

*Other hypothetical proteins with similarity to non-phage proteins*. The 20 CDSs mentioned above showed low but detectable similarity to bacterial (12 CDSs), archaeal (three CDSs), and eukaryotic proteins (5 CDSs) (Table S4). Predicted functions included membrane proteins, lyases, hydrolases, transferases, oxidoreductases, a ligase, an isomerase, a lipid-binding protein, and an RNA-binding protein. Notably, P017 showed low similarity to mitofusin 1 (Identity = 9.6%) and the Smc5/6 complex (Identity = 15.4%), involved in mitochondrial fusion and viral restriction, respectively. The average sequence identity across all hypothetical proteins was 18.9 ± 6.3%, reflecting detection primarily at the level of 3D fold rather than primary sequence.

*Hypothetical proteins with similarity to Bacillota.* AP-20-A harbored 11 CDSs encoding hypothetical proteins showing structural similarity to *Bacillota* proteins (Identity 26.8–79.7%; Coverage 33–98%), with no significant matches to *Alphaproteobacteria* or known rhizobiophages (BLASTp). Among these are P027 (65 aa), which shared 79.7% identity with an unclassified *Peribacillus* protein; P022, which showed 57.3% identity to a *Peribacillus* protein; and P029 (Identity = 48.6%) showing similarity to *Cytobacillus oceanisediminis* (Table S5). These data provide protein-level evidence that AP-20-A shares evolutionary connections with *Bacillota*-infecting podoviruses.

In summary, most hypothetical proteins of AP-20-A are highly divergent and likely represent novel genetic material. However, the detection of similarities to proteins from phages, *Bacillota*, and even archaeal and eukaryotic domains underscores the potential role of extensive horizontal gene transfer in shaping the mosaic genome of this phage.

#### 2.5.3. Protein-Based Clustering of AP-20-A

**Figure 6:**
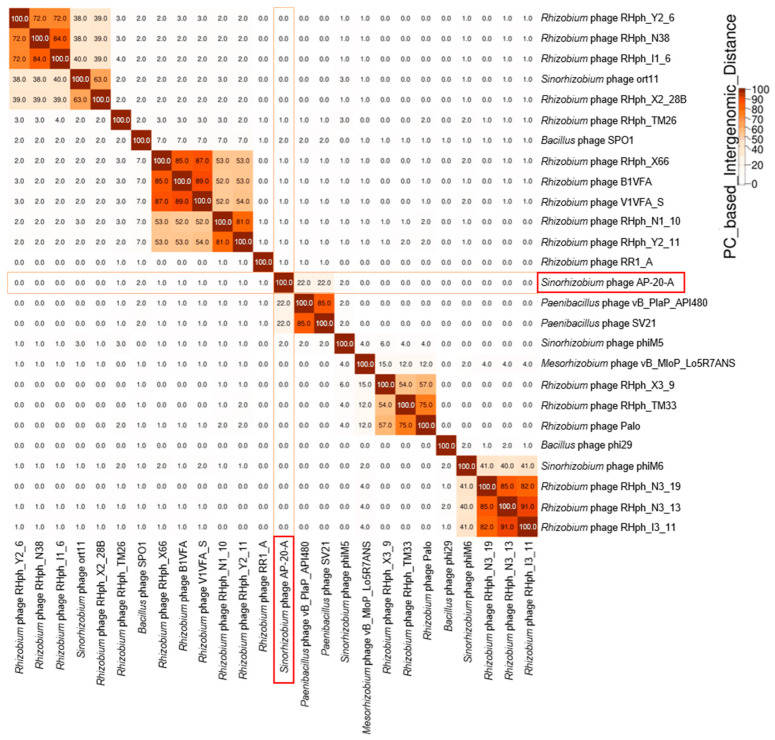
Heatmap of intergenomic similarity of AP-20-A (red outlines) against 25 reference bacteriophage genomes (orange outlines) at the protein cluster (PC) level.

The high proportion (63.6%) of hypothetical and unique proteins underscores the genetic distinctiveness of this phage. This distinctiveness of AP-20-A is quantitatively supported by comparative analysis of its translated complete genome sequence against a comprehensive taxonomically diverse set of 21 rhizobiophage genomes and 4 *Bacillus*- and *Paenibacillus*-infecting phage genomes (see Section 4; Table S6). Using VirClust, intergenomic similarity was detected only with the podoviruses *Paenibacillus* phage SV21 and *Paenibacillus* phage vB_PlaP_API480, reaching 22% in both cases (the similarity between these two phages was 85%; Figure 6). With other bacteriophages, only negligible similarity was detected. Specifically, AP-20-A shared only 2% similarity with the model *Bacillus* phage SPO1 and with the podovirus *Sinorhizobium* phage phiM5, and 1% with seven *Rhizobium* phages (RR1−A, RHph_TM26, RHph_X66, B1VFA, V1VFA−S, RHph_N1_10, RHph_Y2_11; Figure 6). No similarity was detected with the well-characterized podovirus *Sinorhizobium* phage ort11. This profound genomic divergence positions AP-20-A as a unique entity and a promising candidate for discovering novel molecular mechanisms, necessitating detailed phylogenetic analysis to elucidate its evolutionary origins and taxonomic position beyond the current rhizobiophage framework.

#### 2.5.4. Phylogenetic Analysis of AP-20-A

Phylogenetic analyses of phage AP-20-A were performed using three independent datasets: the nucleotide sequences of (1) the complete genome sequences (using VICTOR) (2) the deduced amino acid sequences of the major capsid protein, and (3) the deduced amino acid sequences of the terminase large subunit.

##### Complete Genome Phylogeny

**Figure 7:**
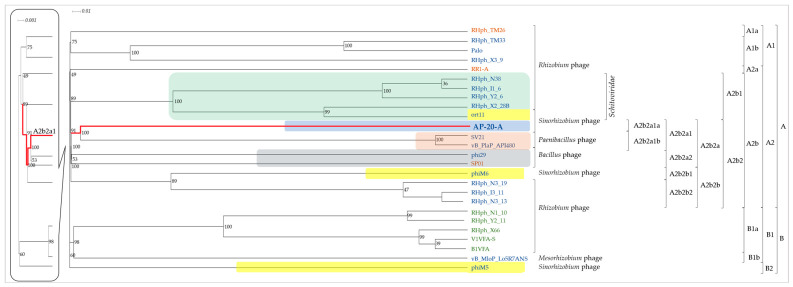
Complete genome phylogenetic analysis of phage AP-20-A. The scale bar indicates 0.1 nucleotide substitutions per site. The tree shows major clades (A, B), subclades (A1, A2, B1, B2), clusters (A2b1, A2b2, B1a, B1b), and subclusters (A2b2a1, A2b2a2, A2b2b1). *Sinorhizobium* phage AP-20-A is highlighted in blue. Podoviruses infecting *Sinorhizobium*—*Sinorhizobium* phage ort11, *Sinorhizobium* phage phiM5, and *Sinorhizobium* phage phiM6—are marked in yellow. Phages belonging to the family *Schitoviridae* are highlighted in green. *Bacillus*-infecting phages are marked in gray, and *Paenibacillus*-infecting phages are marked in orange. Podovirus morphotypes are shown in blue font, myovirus morphotypes in brown font, and siphovirus morphotypes in green font. The branch leading to phage AP-20-A is marked with a red line.

The complete genome sequences of *Sinorhizobium* phage AP-20-A were analyzed together with the same set of 21 rhizobiophages and four phages infecting *Bacillus* and *Paenibacillus* described above (Figure 7). The subsequent division of the A2 subclade revealed that the genome of AP-20-A is phylogenetically affiliated with subclade A2b (bootstrap 89%), which comprises members of the family *Schitoviridae*, including *Sinorhizobium* phage ort11. Within this subclade, AP-20-A clusters with *Paenibacillus* phages (subcluster A2b2a1; bootstrap 100%), which in turn forms a larger group with a cluster containing two *Bacillus*-infecting phages (bootstrap 100%) (Figure 7). This confirms the affiliation of AP-20-A with this lineage while also indicating its distinctness and placement within a phylogenetically distant branch.

##### Phylogeny of Individual Genes

All selected phages mentioned above encode both the major capsid protein (MCP) and the terminase large subunit (TerL).

**Figure 8:**
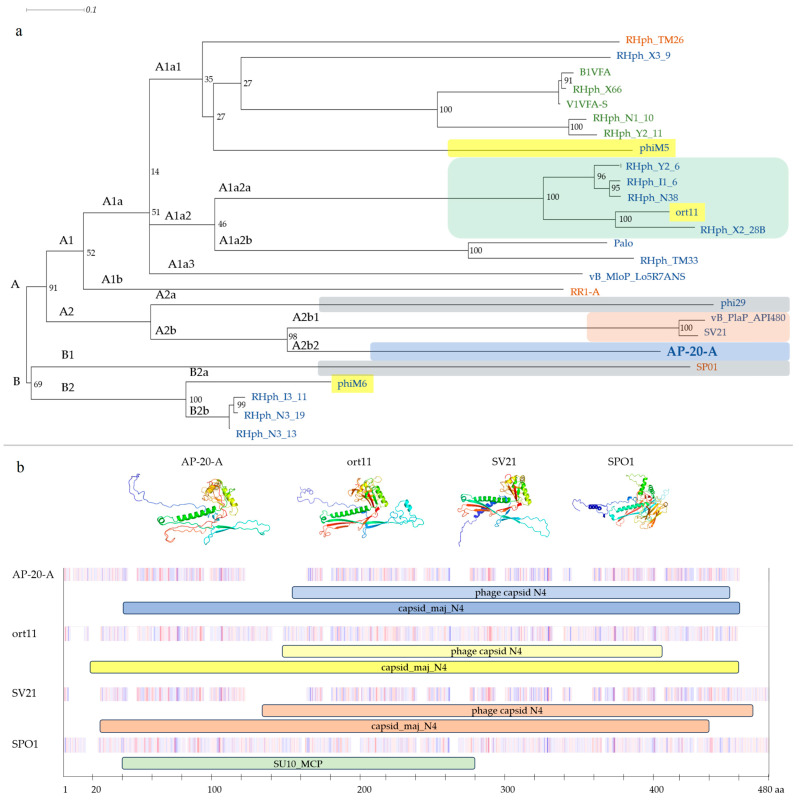
Phylogenetic placement and structural modeling of the major capsid protein of *Sinorhizobium* phage AP-20-A. Panel (**a**) shows a maximum-likelihood phylogenetic tree inferred from multiple sequence alignments. The positions of phage AP-20-A and other *Sinorhizobium* podoviruses (phiM6, phiM5, and ort11) are highlighted. Panel (**b**) presents data from a comparative structural analysis of the MCP of phage AP-20-A. The upper part of the panel shows Richardson diagrams of MCP monomers for phages AP-20-A, ort11, SV21, and SPO1. The lower part shows the amino acid sequence alignment of MCPs with mapped functional domains. The N-terminus is colored from blue to the C-terminus (red). Scale bars for alignments represent 20 amino acids (aa). Sequence alignment with standard “hydro” coloring for amino acid residues (hydrophobic groups). For additional designations and captions, see Figure 7.

*Major capsid protein (MCP, P031)*. Top-down clustering of phylogenetic trees constructed from MCP nucleotide sequences revealed that the closest phylogenetic relative of *Sinorhizobium* phage AP-20-A falls within a broad clade A (bootstrap 91%; Figure 8a) comprising rhizobiophages of the family *Schitoviridae* and *Bacillota*-infecting phages. This clade consists of two subclades, A1 and A2. For the capsid protein, AP-20-A represents a single-member lineage (subcluster A2b2) that merged with cluster A2b1 (bootstrap 98%), which is represented by *Paenibacillus* phage sequences. All of these phages share phylogenetic relatedness to *Bacillus* phage φ29 (cluster A2).

We performed a comparative structural analysis of the primary sequences and predicted tertiary structures of MCPs from AP-20-A, *Sinorhizobium* phage ort11 (a member of the family *Schitoviridae*), *Paenibacillus* phage SV21, and *Bacillus* phage SPO1 (Figure 8b).

The MCP of AP-20-A is 339 aa in length (37.1 kDa, pI 8.87) and shows high similarity to the MCP of SV21 (313 aa, 34.3 kDa, pI 8.87; Identity = 47.44%; Coverage = 97%). Similarity to the MCP of ort11 (400 aa, 43.8 kDa) was lower (Identity = 22.9%; Coverage = 63%) (Figure 8b), while no similarity was detected to the MCP of SPO1 (468 aa, 51.4 kDa, pI 5.51; Figure 8b).

The MCP of phage AP-20-A contains a conserved domain belonging to the N4-gp56 major capsid protein family, as identified using two databases: Pfam (phage_capsid_N4, PF25209) and TIGR (capsid_maj_N4, TIGR04387). However, these domains differ in amino acid composition and length (Figure 8b).

In AP-20-A, the phage_capsid_N4 and capsid_maj_N4 domains consist of 249 and 303 aa, respectively. The MCP domains of AP-20-A contrast markedly with those identified in phage ort11 (family *Schitoviridae*) as well as in phages SV21 and SPO1. In phage SV21, these domains are smaller (216 and 270 aa, respectively), while in ort11 they are larger (299 and 377 aa, respectively).

The phage N4-gp56 domain (PF25209) is a hallmark of certain capsid proteins, including those of *Staphylococcus* phage phiMR11 (A9CRA7) and enterobacterial phage N4 (Q859Q5) [56]. This domain is known to form an icosahedral capsid with T = 9 quasi-symmetry, resulting in a capsid approximately 70 nm in diameter [57], which is consistent with the electron microscopy data for AP-20-A (see Section 2.1).

*Terminase large subunit (TerL, P035)*. The terminase large subunit (TerL) of phage AP-20-A was annotated using two methods: Pharokka and eggNOG-mapper. Two possible overlapping gene variants were identified, with coordinates 32,613–31,231 (1383 bp, p035′) and 32,742–31,231 (1512 bp, p035), respectively. For subsequent analysis, the second gene variant (p035) was used, as its length was most similar to that of the corresponding gene in the model podovirus-morphotype rhizobiophage ort11 (1599 bp).

**Figure 9:**
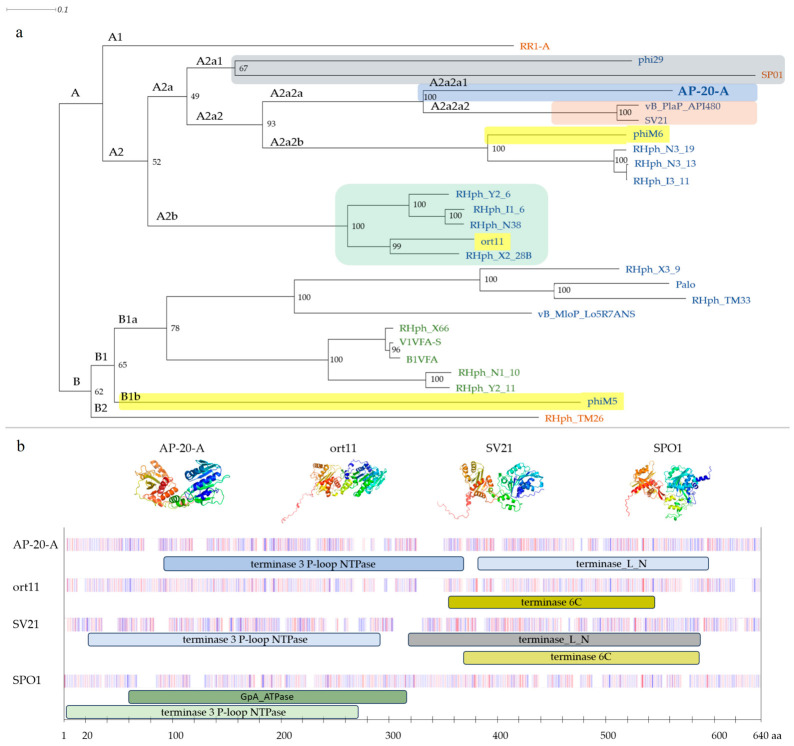
Phylogenetic placement and structural modeling of the terminase large subunit protein of *Sinorhizobium* phage AP-20-A. Panel (**a**) shows a maximum-likelihood phylogenetic tree inferred from multiple sequence alignments of the terminase large subunit (TerL, P035). The positions of phage AP-20-A and other *Sinorhizobium* podoviruses (phiM6, phiM5, and ort11) are highlighted. Panel (**b**) presents data from a comparative structural analysis of the TerL of phage AP-20-A. The upper part of the panel shows Richardson diagrams of TerL monomers for AP-20-A, ort11, SV21, and SPO1. The lower part shows the amino acid sequence alignment of TerLs with mapped functional domains. The N-terminus is colored from blue to the C-terminus (red). Scale bars for alignments represent 20 amino acids (aa). The amino acid sequence alignment uses the same coloring scheme as described for Figure 8. For additional designations and captions, see Figure 7.

Phylogenetic trees constructed from terL nucleotide sequences revealed a broad clade A2 containing subclade A2a, which includes our phage AP-20-A and *Bacillota* phages, and subclade A2b (bootstrap 52%), which includes rhizobiophages of the family *Schitoviridae* (Figure 9a).

Subcluster A2a1 (comprising only two bacillar phages) and subcluster A2a2 have a bootstrap value of 49%. Subcluster A2a2 consists of two further subclusters (bootstrap 93%). One of these, cluster A2a2a, contains our phage AP-20-A (A2a2a1) and two *Paenibacillus* phage sequences (A2a2a2) (bootstrap 100%), whereas cluster A2a2b contains sequences of three *Rhizobium* phages and the podovirus *Sinorhizobium* phage phiM6 (A2a2b; bootstrap 100%) (Figure 9a).

We performed a comparative structural analysis of the primary sequences and predicted tertiary structures of TerL proteins from AP-20-A, *Sinorhizobium* phage ort11 (a member of the family *Schitoviridae*), *Paenibacillus* phage SV21, and *Bacillus* phage SPO1 (Figure 9b). According to this analysis, the TerL protein sequence of AP-20-A (503 aa, 57.81 kDa, pI 5.28) showed 48.7% similarity to the TerL of *Paenibacillus* phage SV21 (455 aa, 52.6 kDa, pI 5.46) with 92% coverage. No similarity was detected to the TerL proteins of rhizobiophage ort11 (532 aa, 61.04 kDa, pI 5.16) or *Bacillus* phage SPO1 (547 aa, 63.7 kDa, pI 7.92).

Domain architecture analysis revealed that the TerL of AP-20-A possesses two domains: a terminase_L_N domain (DNA translocation; IPR035412, according to InterPro) and a terminase 3 P-loop NTPase domain (energy conversion; IPR027417, according to InterPro). A similar domain architecture was found in the TerL of SV21, which additionally contained a terminase_6C domain (IPR035421, according to InterPro), homologous to gp17 of bacteriophage T4 (P17312) [58,59]. The terminase_L_N and terminase 3 P-loop NTPase domains in AP-20-A and SV21 were similar in length but differed in amino acid composition. No similarity was detected between the domains of AP-20-A TerL and those of ort11 or SPO1 (Figure 9b).

Thus, phylogenetic analysis of MCP and TerL sequences places AP-20-A within a clade containing *Paenibacillus* and *Bacillus* phages, rather than within the *Schitoviridae* family of rhizobiophages. The MCP of AP-20-A shares the N4-like domain architecture with both SV21 and ort11 but differs in domain lengths. The TerL of AP-20-A clusters robustly (100% bootstrap) with two *Paenibacillus* phages and shows no detectable similarity to the TerL of rhizobiophage ort11 or *Bacillus* phage SPO1. Thus, AP-20-A represents a novel *Sinorhizobium* phage lineage with phylogenetic affinities to *Bacillota*-infecting phages, while maintaining strict host specificity for *Sinorhizobium*.

## 3. Discussion

*Sinorhizobium* phage AP-20-A is an obligately lytic podovirus (93%, PhageGE) with negligible genomic synteny to known rhizobiophages (<2%). Its core structural proteins show closest homology to podoviruses infecting *Bacillota*, including *Bacillus* and *Paenibacillus*. This is supported at the protein level: multiple AP-20-A proteins share highest similarity exclusively with *Bacillota* (*Bacillus*, *Peribacillus*, *Paenibacillus*, *Cytobacillus*), with amino acid identities reaching 79.7% (coverage up to 98%). No significant matches were detected to *Alphaproteobacteria* or known rhizobiophages. This is consistently observed at the protein level.

This phylogenetic link suggests that the soil virome may function as a genetic reservoir enabling HGT between *Bacillota* and *Alphaproteobacteria*, a phenomenon not previously documented in rhizobiophages to our knowledge. Horizontal transfer between phages is possible, as shown by conserved RBD motifs in *Kuttervirus* and *Gamaleyavirus* facilitating recombination and host switching [60]. Another example is a group of promiscuous podophages whose hosts belong to different *Gammaproteobacteria* genera [61]. This cross-phylum exchange is ecologically plausible: *Paenibacillus* and *Sinorhizobium* co-exist in root rhizosphere [62]. Acquisition of a structural module from a *Paenibacillus*-infecting phage may have allowed the ancestor of AP-20-A to acquire a novel host recognition system, a potential step toward specialization on *Sinorhizobium*.

AP-20-A combines several features not previously observed together in a lytic genome: interphylum origin of structural genes, a DNA packaging module containing an FtsK/SpoIIIE family protein (rarely reported in podoviruses), and structural homology of several phage proteins to bacterial enzymes. These characteristics place AP-20-A outside the current taxonomic framework of rhizobiophages.

If AP-20-A acquired its structural core from a *Bacillus* phage, a recent host jump might be considered. However, two molecular features argue against this. First, the phage encodes no tRNAs, suggesting long term translational compatibility with *S. meliloti* [63]. Second, its codon usage shows dual adaptation (host-abundant and host-rare codons), a degree of fine-tuning difficult to reconcile with a recent host shift. As shown for dsDNA phages, codon adaptation correlates with co evolutionary history [64,65,66]. Thus, AP-20-A’s genome may reflect a two-stage history: HGT acquisition of a structural module, followed by extensive optimization for replication in *S. meliloti*.

A characteristic genomic feature is the abundance of hypothetical proteins (63.6% of CDSs), consistent with genome wide observations that about 38% of phage ORFs are ORFans contributing to phage diversity [67,68]. For most, similarity was detectable only at the level of 3D structural folds (average identity 18.9%). One exception is P015, which encodes a Tad2-like domain, a component of the Thoeris type II anti-defense system blocking abortive infection [55]. The presence of a Tad2 protein in a rhizobiophage has not been reported previously; whether Tad2 confers anti Thoeris activity remains to be determined.

The resistant strain AK555 harbored an additional AbiE system absent in the sensitive strain Md3/4, while Md3/4 carried more CRISPR-Cas cassettes that did not target AP-20-A. This suggests that AbiE may contribute to resistance, whereas R-M and CRISPR are unlikely to be primary determinants. Adaptive CRISPR systems require prior exposure to acquire spacers, whereas abortive infection systems confer immediate innate resistance. The absence of AP-20-A-targeting spacers in Md3/4 suggests that this phage is evolutionarily novel to these hosts, while the additional AbiE system in AK555 may have provided effective immediate defense. This observation suggests that AbiE systems, being non-adaptive and capable of targeting a broad range of phages without prior co-evolution [69,70], may be particularly effective against novel viruses. In contrast, CRISPR-Cas immunity requires matching spacers, which may explain why Md3/4—despite more cassettes—remained susceptible. Tad2 of the phage and AbiE represent independent defense–counterdefense layers; their potential interplay in phage-bacteria interaction requires further investigation.

The lytic activity of AP-20-A is ecologically patterned. Among susceptible native strains, 60% entered a state consistent with putative pseudolysogeny (the genome lacks integrase and repressor genes). Strain Md3/4 exemplified an extreme case: infection led to a prolonged growth stimulation (final density 1.45× control). This aligns with large scale analyses suggesting that carrier like states may be widespread among phages traditionally classified as strictly lytic [71,72]. Putative pseudolysogeny thus represents a third ecological outcome buffering host populations while maintaining phage dissemination.

Resistance to AP-20-A is ecologically stratified. Agrocenose (agricultural) soil isolates showed higher resistance frequency than phytocenose isolates (P_F_ = 0.036), suggesting that cultivation history correlates with increased resistance. Recent work has shown that the Hna defense system in *S. meliloti* undergoes extensive horizontal transfer and is modulated by host factors such as NolR [73]. Consequently, elite inoculant strains naive to local phages may lack co-evolved defenses and could be particularly vulnerable.

From a biotechnology perspective, the intrinsic resistance of industrial *S. meliloti* strains (Eff^++^) to AP-20-A, combined with its lytic activity against susceptible native isolates, makes the phage a candidate for inclusion in phage cocktails. Recent studies have shown that phage cocktails outperform single phages in reducing bacterial pathogen loads while largely sparing beneficial rhizosphere bacteria [7]. The differential susceptibility we observed (resistant native strains versus vulnerable elite inoculants) provides a functional rationale for “phage-assisted inoculant delivery,” a concept proposed but rarely experimentally validated. Unlike the use of phages as typing tools [34], our study positions AP-20-A as an agent that distinguishes between phage-experienced (native) and phage-naive (inoculant) populations. This suggests that inoculant efficacy may depend on the local viral context, and that assessment of resident phage communities could become integral to inoculant development—a conclusion that aligns with recent calls to integrate soil virome assessment into microbiome management strategies [7].

Several open questions remain. The molecular basis of resistance in agrocenose strains is unknown. The molecular switch controlling lysis, putative pseudolysogeny, or resistance is not yet understood. Functional characterization of orphan proteins beyond Tad2 would be required to fully understand their roles; several hypothetical proteins contain predicted transmembrane domains and could represent novel lysis or receptor binding proteins.

In conclusion, *Sinorhizobium* phage AP-20-A is an unusual lytic podovirus whose structural core resembles phages of *Bacillota*, not known rhizobiophages. Its genome is consistent with a two-stage history: cross-phylum gene transfer followed by codon adaptation. The presence of a Tad2-like domain offers a target for future investigation. The observation of putative pseudolysogeny in most susceptible native strains adds complexity to phage–host interactions in soil. Managing soil microbiomes for agriculture may benefit from considering the local phage community.

## 4. Materials and Methods

### 4.1. Phages Isolation and Purification

*Sinorhizobium* phage AP-20-A was isolated from a 5 g soil surface sample (5–10 cm) [74,75] collected from an agricultural field under green manure fallow in the Voronezh region, Russia. The soil of the experimental site is classified as Calcic Chernozem (heavy loam, low humus content) with a humus content of 3.5% and a neutral pH of 7.2 [76,77]. The phage was isolated according to a previously described enrichment protocol [36], using a set of nine phage-sensitive *Sinorhizobium meliloti* strains grown in LB medium as described by Barnet [78].

### 4.2. Bacterial Strains and Growth Conditions

A total of 78 native *S. meliloti* strains were randomly selected from the working collection of the Laboratory of Genetics and Selection of Microorganisms at FSBSI ARRIAM. Species identification had been previously confirmed by PCR-RFLP analysis [79]. The *S. meliloti* strains set comprised both nodule (N) and soil (S) isolates obtained either by the plant trapping method under laboratory conditions or directly from nodules of wild alfalfa plants using the method described in [32]. To ensure representativeness, isolates were collected from several geographically distant phytocenoses and agrocenoses. The final sample set was represented by three groups: soil (PS) and nodule (PN) isolates from phytocenoses (32 and 18 strains, respectively), and soil isolates from agrocenoses (AS, 28 strains). No significant differences in the proportion of sensitive and resistant isolates were detected among strains from different locations within the same habitat type (Fisher’s exact test, P_F_ > 0.05), justifying their pooling into the respective ecological groups. The overall strain panel was balanced (E = 0.97) and heterogeneous (H = 1.07).

Additionally, four highly efficient (Eff^++^) strains of *S. meliloti*, five strains of *S. medicae*, and the strain *S. fredii* DSM5851 were tested. To assess the host specificity of phage AP-20-A beyond the *Sinorhizobium* genus, a panel of nine non-rhizobial bacterial strains was included. This panel comprised several species of the phylum *Bacillota*, given the genomic similarity of AP-20-A to *Bacillus*-infecting phages, as well as representative *Agrobacterium* and *Escherichia coli* strains. The non-rhizobial strains were: *Agrobacterium radiobacter* strain 204 (*A. tumefaciens*) (from the laboratory collection, see above), *A. fabrum* C58 (from the Russian Collection of Agricultural Microorganisms, RCAM, St. Petersburg, Russian Federation; http://www.arriam.ru/kollekciya-kul-tur1/; accessed on 1 June 2025), *E. coli* DH5α, *Bacillus thuringiensis* 800/15, *B. mycoides* b12.3, *Lysinibacillus sphaericus* 1795, and *Peribacillus frigoritolerance* d21.2 (from the collection of the Laboratory of Proteomics of Supra-organizational Systems, FSBSI ARRIAM).

Tested strains were cultured on LA (2% agar) and LB broth media at 28 °C. Liquid cultures were grown with shaking at 180 rpm. Optical density was measured at OD_600_ using a spectrophotometer. For experiments on phage–microbial interactions (host range, efficiency of plating (EOP), one-step growth experiment, and dynamics of phage–microbial interactions), 16-h cultures of bacterial strains were used.

### 4.3. Evaluation of Phage Host Range

The host range (lytic activity) of phage AP-20-A was evaluated using a spot test assay, as previously described [25], against 78 native and 4 Eff^++^ *S. meliloti* strains and 9 non-rhizobial strains mentioned above. The degree of spot lysis was assessed as follows: (1) a transparent spot with a clear edge was considered as complete lysis; (2) a cloudy and opaque spot with an unclear edge was considered as incomplete lysis; (3) the absence of a lysis spot was considered as no lysis (strain resistance). Significant differences were assessed using Fisher’s exact test (P_F_).

The Efficiency of Plating (EOP) was determined using a spot test assay with a series of ten-fold dilutions of the phage stock/phage lysate. The result was evaluated as the average plaque-forming unit (PFU) count on strains with positive spot test results divided by the average PFU count on the lab host strain, as previously described [80]. For each phage–bacterium combination, the obtained EOP values were assigned to the following groups: “High production” when the ratio was ≥0.5, indicating that infection of the target bacterium yielded at least 50% PFU/mL relative to the host strain. “Medium production” if the EOP was ≥0.1 but <0.5. “Low production” if the EOP was >0.001 but <0.1. “Inefficient” if the EOP was ≤0.001 [81,82].

### 4.4. One-Step Growth Curve of Phage AP-20-A

Phage AP-20-A’s latent period and burst size were estimated according to the protocol described in [83], with some modifications: the *S. meliloti* strain AT was grown at 28 °C and the total run time of the experiment was 260 min.

### 4.5. Evaluation Dynamics of Phage–Microbial Interactions

Phage–microbial interactions were analyzed based on changes in bacterial growth curves and the multiplicity of infection (MOI). The MOI, defined as the ratio of the number of virions to the number of bacterial cells at the start of the experiment [84], was set at two values: 0.005 and 0.0006. These MOI values corresponded to approximately 40,000 and 5000 viral particles, respectively. Phage particles were enumerated using the double-layer agar method [85,86], and bacterial cell counts were determined using a QUANTOM Tx Microbial Cell Counter (Logos Biosystems, Anyang-si, Gyeonggi-do, Republic of Korea).

The dynamics of phage–microbial interactions were studied using three *S. meliloti* strains: fully sensitive host-strain AT, phage-sensitive strain Md3/4 and phage-resistant strain AK555. *S. meliloti* strain AK555 was isolated from a nodule of wild-growing *Medicago falcata* plant from the Shalkar District (Mugodzhar mountain region) in northern Kazakhstan, which belongs to the modern center of introgressive hybridization of alfalfa species. Its genome’s characteristics were described in [87].

*S. meliloti* strain AT was isolated from a soil sample from the Shalkar District, using the trapping method with *M. truncatula*. *S. meliloti* strain Md3/4 was isolated from soils of the mountainous area of the center of diversity of cultivated plants in the Caucasus, using the trapping method with *M. varia*. Preliminary whole-genome sequencing data revealed that the two strains differ in the sizes of their main replicons and the number of cryptic plasmids. For strain AT, the chromosome size is 3.71 Mb, pSymA is 1.92 Mb, pSymB is 1.66 Mb, and three cryptic plasmids were detected. For strain Md3/4, the chromosome size is 3.61 Mb, pSymA is 1.60 Mb, pSymB is 1.59 Mb, and one cryptic plasmid was detected. The genomes also differ in the number of phage-derived sequences (8 in AT, 4 in Md3/4). Analysis of anti-phage defense systems was performed using the PADLOC web server [88] and the CRISPR/Cas Finder v1.1.0 tool [89], integrated into Proksee. Previous studies have also shown that these strains exhibit differential sensitivity to other bacteriophages [25,80].

The experiment was conducted in quadruplicate in LB broth with initial OD_600_ = 0.106 ± 0.012 of described strains over 40 h (28 °C) using a Synergy H1 microplate reader (Bio-Tek Instruments Inc., Winooski, VT, USA). Measurements were taken every 10 min with preliminary orbital shakes.

### 4.6. Transmission Electron Microscopy

Phage particles were examined by negative contrast method, as previously described [25]. Briefly, the suspension was adsorbed onto 300-mesh copper grids (Sigma-Aldrich, St. Louis, MO, USA) coated with a collodion support film. To produce a supporting film on electron microscopy grids, we used a 2% collodion solution in amyl acetate (cat. num. 09817, 9004-70-0 Sigma-Aldrich). To prepare the film, a drop of 2% collodion solution was dropped onto the surface of distilled water. After evaporation of the amyl acetate, a thin collodion film formed on the surface of the water. EM grids (300 mesh) were placed on this film. Then, grids with film were picked-up on a piece of parafilm. After 1–2 min of adsorption, the grids were washed twice with distilled water and subsequently negatively stained with a 2% solution of sodium phosphotungstate (Sigma-Aldrich, St. Louis, MO, USA, pH 7.2) for 1–2 min. The samples were then dried and examined using a JEM 1011 transmission electron microscope (JEOL, Tokyo, Japan). Micrographs were acquired with a Morada high-resolution digital camera (Olympus, Tokyo, Japan) at instrumental magnifications ranging from 60,000× to 250,000×.

### 4.7. DNA Isolation, Sequencing, Assembly, and Annotation of the Bacteriophage Genome

Genomic DNA of phage AP-20-A was isolated using the GeneJET Viral DNA/RNA Purification Kit (Thermo Fisher Scientific, Waltham, MA, USA). Genome sequencing was performed as described previously [25] at the Genomics Center for Collective Use of the Siberian Branch of the Russian Academy of Sciences (Novosibirsk, Russia). De novo assembly was performed using SPAdes v4.0.0 with filtered reads [90,91].

Phage genome annotation was performed using eggNOG v7.0 [92], Pharokka v1.9.1 [93] and PHROG (Prokaryotic Virus Remote Homologous Groups) [94]. To predict the position of the replication origin, the Ori-Finder 2022 software was used (https://tubic.org/Ori-Finder, accessed on 17 December 2025; [95]). GC composition variations were determined by GC skew using GenSkew v1.0. A physical map of the phage AP-20-A genome was generated using the Proksee web server (https://proksee.ca/, accessed on 17 December 2025). The lifestyle of phage AP-20-A was predicted bioinformatically using two complementary online platforms: the automated analysis pipeline of the PhageScope v1.3 database [96] and the integrated web server PhageGE [97]. Alien Hunter (v1.3.0) integrated into Proksee, was used to predict potential horizontal gene transfer (HGT) events based on atypical nucleotide composition indicative of foreign DNA acquisition [98].

Analysis of 42 hypothetical CDSs was performed using InterProScan, SWISS-MODEL web server [99] and BLASTp. The SWISS-MODEL and I-TASSER web servers were used to predict three-dimensional structural homologs for each of these CDSs by comparison with experimentally determined structures from the Protein Data Bank (PDB), AlphaFoldDB, and the SWISS-MODEL repository. Model quality was assessed using the Global Model Quality Estimate (GMQE), which ranges from 0 to 1 (higher values indicate greater reliability).

### 4.8. Selection of Rhizobiophage Genomes for Comparative Analysis

A total of 201 rhizobiophage genomes were available in GenBank, including 20 phages infecting *Agrobacterium* spp., 142 infecting *Rhizobium* spp., 12 infecting *Bradyrhizobium* and *Mesorhizobium* spp., and 27 infecting *Sinorhizobium* spp. From these, a set of 21 rhizobiophage genomes was selected for comparative analysis. The selection criteria included genome identity below 95% (to ensure representation of different genera [100,101]), the presence of annotated genes encoding the terminase large subunit and the major capsid protein, and a preference for phages belonging to different families, exhibiting different virion morphologies, and infecting various rhizobial species. All genomes were retrieved from the NCBI database on 3 October 2025 (see Table S6). This set included three podoviruses (phiM6, phiM5, and ort11) infecting *Sinorhizobium* spp. [28,37,102] and 18 other rhizobiophages [103,104,105,106,107], which were identified in preliminary Mauve and VirClust analyses as sharing minimal similarity with AP-20-A (all genomes were retrieved from the NCBI database on 3 October 2025; see Table S6). Additionally, the genomes of four *Bacillus*-infecting phages (*Bacillus* phage φ29, *Bacillus* phage SP01, *Paenibacillus* phage SV21, and *Paenibacillus* phage vB_PlaP_API480, [108,109,110,111]; Table S6), were included in the analysis, as similarity to these phages was detected at the level of individual CDSs.

### 4.9. Nucleotide and Amino Acid Sequence Analysis

Nucleotide and amino acid sequences were analyzed for similarity using BLAST programs against the NCBI non-redundant database (accessed on 1 August 2025) [112]. Codon usage frequencies were determined using the formula: F(c) = N(c)/A, where F(c) is the frequency of a specific codon in the phage genome, N(c) is the total number of codons in all phage genes, and A is the total number of all codons for the amino acid to which the codon corresponds, across all phage genes. The number of specific codons in each phage gene was determined using Sequence Manipulation Suite: Codon Usage [113]. For *S. meliloti*, codon usage frequencies were determined based on the nucleotide sequences of 120 core genes [25,114,115]. Heatmaps of protein clusters were constructed based on hierarchical clustering of viral genomes using VirClust v2.0 [116]. Sequence alignment of 26 phage genomes was performed using Mauve v20150226 build 10 [117]. The primary protein sequences were aligned, and their biochemical properties (Isoelectric point (pI), molecular weight) were predicted using Benchling (https://benchling.com; accessed on 17 December 2025). Functional domains, their characteristics, and coordinates were predicted using InterPro (accessed on 16 December of 2025; [118]). Three-dimensional protein structure models were predicted using the I-TASSER web server [119,120] and SWISS-MODEL [99] and visualized using PyMOL v3.1.4.1 (http://www.pymol.org/pymol (accessed on 2 April 2026)).

### 4.10. Phylogenetic Analysis

Complete genome sequences phylogenetic analysis of the phages was performed using the VICTOR web service (https://victor.dsmz.de, accessed on 1 November 2025) for genome-based phylogeny and classification of prokaryotic viruses [121]. All pairwise comparisons of nucleotide sequences were conducted with the Genome-BLAST Distance Phylogeny (GBDP) method [122] using settings recommended for prokaryotic viruses [121]. The resulting intergenomic distances were used to infer a balanced minimum evolution tree with branch support via FASTME, including SPR postprocessing [123] for the D0 formula. Branch support was assessed from 100 pseudo-bootstrap replicates.

Multiple sequence alignment of the major capsid protein, terminase large subunit and Tad2 protein was performed using MUSCLE [124]. Amino acid residues in the alignments shown in Figure 5, Figure 8 and Figure 9 are colored according to the standard “hydro” hydrophobicity-based scheme. Phylogenetic trees were constructed with IQ-TREE version 1.6.7 [125] using the maximum likelihood algorithm with 1000 bootstrap replicates. The ModelFinder option was used to select the best-fit substitution models, resulting in the TN+F+G4 model for the major capsid protein and the GTR+F+I+G4 model for the terminase large subunit [126]. All trees were midpoint-rooted [127] and visualized in Dendroscope 3 [128].

### 4.11. Statistical Analysis

Statistical methods appropriate for small sample sizes were used in this study. Comparative analysis of different groups was performed using Fisher’s exact test (significance level, α = 0.05). Confidence intervals were determined according to the Clopper–Pearson method using PAST v. 4.03 software (Oslo, Norway) [129].

### 4.12. Nucleotide Accession

The genome sequence of phage AP-20-A has been deposited in GenBank under accession number PX794722.

## Figures and Tables

**Table 1:** Genomic characteristics of *Sinorhizobium* phage AP-20-A.

Genome	*Sinorhizobium* Phage AP-20-A
Genome size, kbp	49.4
G + C, %	43.1%
tRNA	-
Total CDS ^1^:	66
Proteins with assigned function (Number, % of total CDS)	24 (36.4%)
Phage particle morphogenesis proteins	5 (7.6% ^2^)
Enzymes (DNA metabolism, phage-host takeover and metabolic modulation, etc.)	19 (28.8% ^2^)
Hypothetical proteins (Number, % of total CDS)	42 (63.6%)
With database hits ^3^	40 (60.6% ^2^)
Unique proteins (no hits)	2 (3% ^2^)

“-”—sequences not identified; ^1^—annotated with eggNOG and Pharokka; ^2^—percentage of CDSs per functional group (%); ^3^—according to SWISS-MODEL and BLASTp.

**Table 2:** Analysis of core structural and packaging module genes and their products in phage AP-20-A.

AP-20-A Gene/CDS Number According to Pharokka ID	Length, aa	Predicted Function (PHROG)	Sequences Producing Significant Alignments (BLASTp)			The Closer Structural Analog (I-TASSER Web Server)			
			Best Match (Protein)	Source	Identity/Coverage, %	Best Match (Protein)	Source (PDB Accession)	Identity/Coverage, %	3D Model
**Structural proteins**									
**p019**	283	Lytic tail fiber protein (14342)	Phage tail tape measure protein	*Priestia aryabhattai*	67.20/43	antimicrobial peptidase lysostaphin	*Staphylococcus simulans* (4LXC)	20.3/82.0	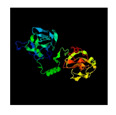
**p024**	558	Head closure Hc3 (578)	Head closure Hc3	*Paenibacillus* phage vB_PlaP_API480	36.66/91	head-to-tail adapter	*Pseudomonas* phage Pa223 (9NWI)	13.75/22	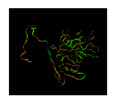
**p031**	339	Major head protein (1357)	N4-gp56 family major capsid protein	*Psychrobacillus* sp.	53.31/89	Capsid asymmetric unit	Cyanophage Pam1 (7EEL)	13.2/90.6	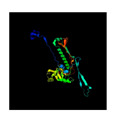
**p033**	629	Portal protein (156)	Portal protein	*Paenibacillus larvae*	44.81/94	Portal Protein	*Lederbergvirus* P22 (3LJ5)	10.1/93.5	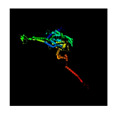
**p036**	819	Tail protein (18629)	Tail protein	*Pagevirus* palmer	39.60/18	arabinanase	*Geobacillus stearothermophilus* (5HO0)	12.3/88.6	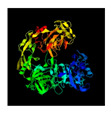
**Packaging module**									
**p035**	503	Terminase large subunit (2)	Terminase large subunit	*Paenibacillus* phage vB_PlaP_API480	47.06/98	Large subunit terminase	*G. stearothermophilus* D6E (5OE8)	16.49/76.0	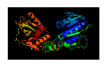
**p011**	425	FtsK/SpoIIIE family protein (1358)	FtsK/SpoIIIE family protein	*Caudoviricetes* sp.	35.37/92%	DNA translocase FtsK	*E. coli* BL21 (DE3) (2IUS)	20.65/80.0	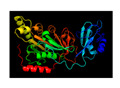

## Data Availability

The genome sequence of AP-20-A was deposited in GenBank with the nucleotide accession number PX794722.

## Supplementary Materials

The following supporting information can be downloaded at https://www.mdpi.com/article/10.3390/ijms27125515/s1.
