# Supplementary figures and images for: A Novel Lytic Podovirus AP-20-A Infecting Sinorhizobium meliloti: Mosaic Genome with Cross-Phylum Homology and Implications for Inoculant Establishment
Source: Int J Mol Sci. 2026 Jun 18;27(12):5515. doi: 10.3390/ijms27125515 (PMC13299392; doi:10.3390/ijms27125515)

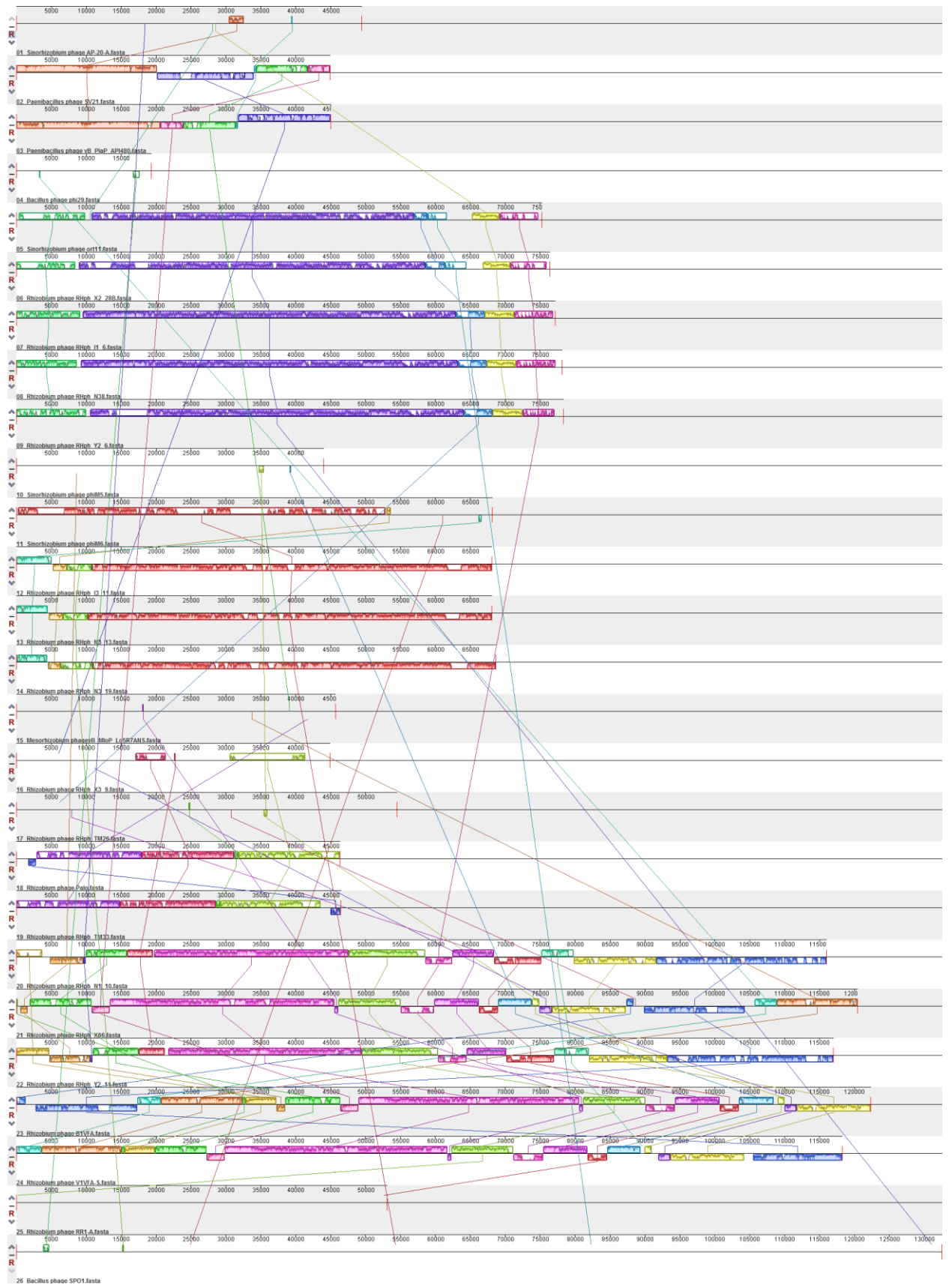

Figure S1: The Mauve alignment of AP-20-A against 25 reference bacteriophage genomes.

Supplement: Supplementary file 1 [file ijms-27-05515-s001.zip › Figure S1.pdf]
